# HYBRIDdb: a database of hybrid genes in the human genome

**DOI:** 10.1186/1471-2164-8-128

**Published:** 2007-05-23

**Authors:** Dae-Soo Kim, Jae-Won Huh, Heui-Soo Kim

**Affiliations:** 1 PBBRC, Interdisciplinary Research Program of Bioinformatics, College of Natural Sciences, Pusan National University, Busan 609-735, Korea; 2 Division of Biological Sciences, College of Natural Sciences, Pusan National University, Busan 609-735, Korea

## Abstract

**Background:**

Hybrid genes are candidate risk factors for human tumors by inducing mutation, translocation, inversion, or rearrangement of genes. The occurrence of hybrid genes may also have given rise to new transcripts during hominid evolution.

**Description:**

HYBRIDdb is a database of hybrid genes in humans. This system encompasses the bioinformatics analysis of mRNA, EST, cDNA, and genomic DNA sequences in the INDC databases, and can be used to identify hybrid genes. We searched for hybrid genes among the 28,171 genes listed in the NCBI database, and analyzed their structural patterns in the human genome. The 2,344 gene pairs were detected as hybrid forms of transcriptional products. We classified the hybrid genes into two groups: chromosomal-mediated translocation fusion transcripts and transcription-mediated fusion transcripts.

**Conclusion:**

The HYBRIDdb database will provide genome scientists with insight into potential roles for hybrid genes in human evolution and disease.

## Background

Hybrid genes are created by trans-splicing, sense/antisense transcription, genome rearrangement, or intergenic splicing between two genes [1-5]. The creation of new genes is a potential risk factor for tumor development, yet may also promote diversification by inducing gene substitution, translocation, inversion, or rearrangement [1,6-8]. As a result, hybrid genes have the ability to be either harmful or advantageous to humans [9,10].

Cancer genes have somatic mutations similar to chromosomal translocations that result in hybrid transcripts by apposing one gene to the regulatory regions of another. For example, a hybrid transcript is created by genomic rearrangement of the mixed-lineage leukemia (MLL) gene at 11q23 and the septin family (SEPT6) gene at Xq24 in acute leukemia patients [11]. These rearrangements result in fusions with at least 40 other genes, resulting in expression of hybrid proteins with leukemogenic activity [12,13]. Occasionally hybrid gene formations can also contribute to genomic diversity. Normal forms of *UEV *proteins are located in the nuclei of cells, while *Kua *proteins are distributed in endomembranes. The abnormal fusion transcript of these proteins, however, has new enzymatic activity that is associated with cytoplasmic structures [9,14,15]. Thus, *UEV-Kua *gene fusion may be a critical step toward creating a protein with a novel function. The result of splicing out the intergenic region is reported to be a new mechanism of intergenic splicing [15,17]. For example, the *HHLA1-OC90 *fusion transcript is expressed by a heterologous HERV-H LTR promoter that is highly active in a teratocarcinoma cell line [18]. Intergenic splicing also occurs between the *SSF1 *and *P2Y*_11 _genes on human chromosome 19 [19]. The *SSF1-P2Y*_11 _gene fusion product is 5.6 kb mRNA in length and results in the addition of a potential ATP binding site in *SSF1 *[19]. Trans-splicing joins two independently transcribed mRNA sequences at canonical exon-exon borders. Although this is a wide- spread phenomenon among lower eukaryotes, only a few isolated cases have been reported in mammals. In the hybrid *CYP3A *transcripts, *CYP3A43 *exon 1 is joined to distinct sets of *CYP3A4 *or *CYP3A5 *exons at canonical splice sites [5].

Although advanced cytogenetic banding experiments reveal important information about gene rearrangement mechanisms, experimental screening is costly, time consuming, and tedious. The translocation-mediated hybrid transcript using bioinformatic tools was examined [20,21]. Kapranov et al. [14], Parra et al. [15], and Akiva et al. [16] have also analyzed the intergenic splicing-mediated gene fusion mechanism only in the human genome. Therefore, we describe the database, HYBRIDdb, which was designed to detect all of the human hybrid genes (chromosomal-mediated translocation, intergenic splicing-mediated, and few trans-splicing hybrid genes) from publicly available transcript sequences for the understanding of the complex gene catalog in normal and abnormal human tissues. We systematically identified hybrid genes from human sequences and discovered some unique features. Included in this database is a comprehensive list of hybrid genes created by trans-splicing, intergenic splicing, and genomic rearrangement between two human genes.

## Construction and content

### Data set

The human transcript (mRNA, EST, cDNA) and human genome sequences were downloaded from the NCBI database. Mobile elements in the human genome sequences were identified by RepeatMasker [22], and transposable element consensus sequences were identified by Repbase Update [23]. Useful transcript information from tissues and pathology samples was obtained from NCBI genbank.

### *In silico *identification of transcriptional hybrid genes

**Figure 1:**
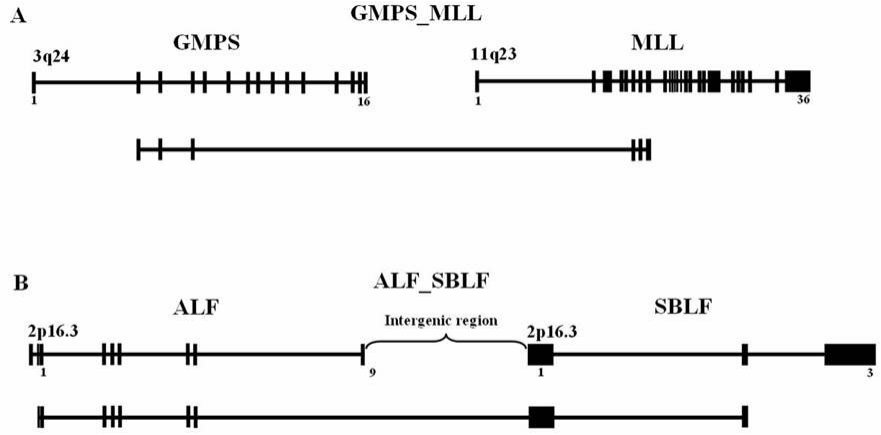
Schematic representation of transcriptional hybrid gene. A model for transcriptional hybrid gene. The hybrid genes, GMPS_MLL(A) and ALF_SBLF (B), are represented. GMPS_MLL hybrid transcript was created by genome rearrangement. ALF_SBLF was a hybrid transcripts containing exons intergenic splicing between two genes. Boxes represent the exons, and bold line indicates the introns.

To characterize the phenomenon of hybrid genes present in the human genome, we clustered RefSeq mRNA onto the human genome sequence (NCBI Build 35.1) using the SIM4 program. RefSeq mRNA was obtained from the NCBI Genbank database. If RefSeq mRNA sequences overlapped, only the longest was considered. After aligning the mRNAs using the SIM4 program [24], we removed naturally overlapping gene pairs based on the coordinates of RefSeq in the human genome sequence. We filtered out connecting sequences with high scoring alignment in both genes. Then, human mRNA, cDNA, and EST sequences in the NCBI GenBank were aligned to human RefSeq using the MegaBLAST program [25]. Alignments having >97% sequence identity and a minimum length of 100 bp were used in this study. We searched hybrid transcript sequences having high-scoring alignments in both genes. To remove the cDNA and mRNA sequences which were suspected to have DNA contaminations, we selected the sequences which have minimum two different sources of transcript (mRNA, cDNA, EST). And also they should be spliced canonically and share at least one splice site with each of the two separate genes. We extracted the human transcript sequences (mRNA, cDNA, EST) which were aligned to two different RefSeq mRNA sequences. For the confirmation of hybrid transcript candidate, we aligned our candidate sequences to genomic DNA sequence by using the SIM4 program [24] and the alignment around the fusion point was manually inspected. To discard the false-positive results of alignment error derived by the homologous gene family, we filtered out connecting sequences having high-scoring alignments in both genes. We extracted the position information of the exon and genome sequences to be matched. Based on this information, the location of the hybrid transcripts and exons were calculated from their position on the genome. We searched for canonically spliced sequences connecting these two transcripts that share at least one splice site with each of the genes. To avoid the use of DNA-contaminated cDNA sequences, we demanded that the sequences connecting these two transcripts should be canonically spliced and share at least one splice site with each of the two separate genes. This procedure identified 2,344 pairs of the 28,171 genes as hybrid transcript candidates, including hybrids transcript created by chromosomal translocation (Figure 1A) or intergenic splicing between two genes (Figure 1B).

## Utility and discussion

### User interface

**Figure 2:**
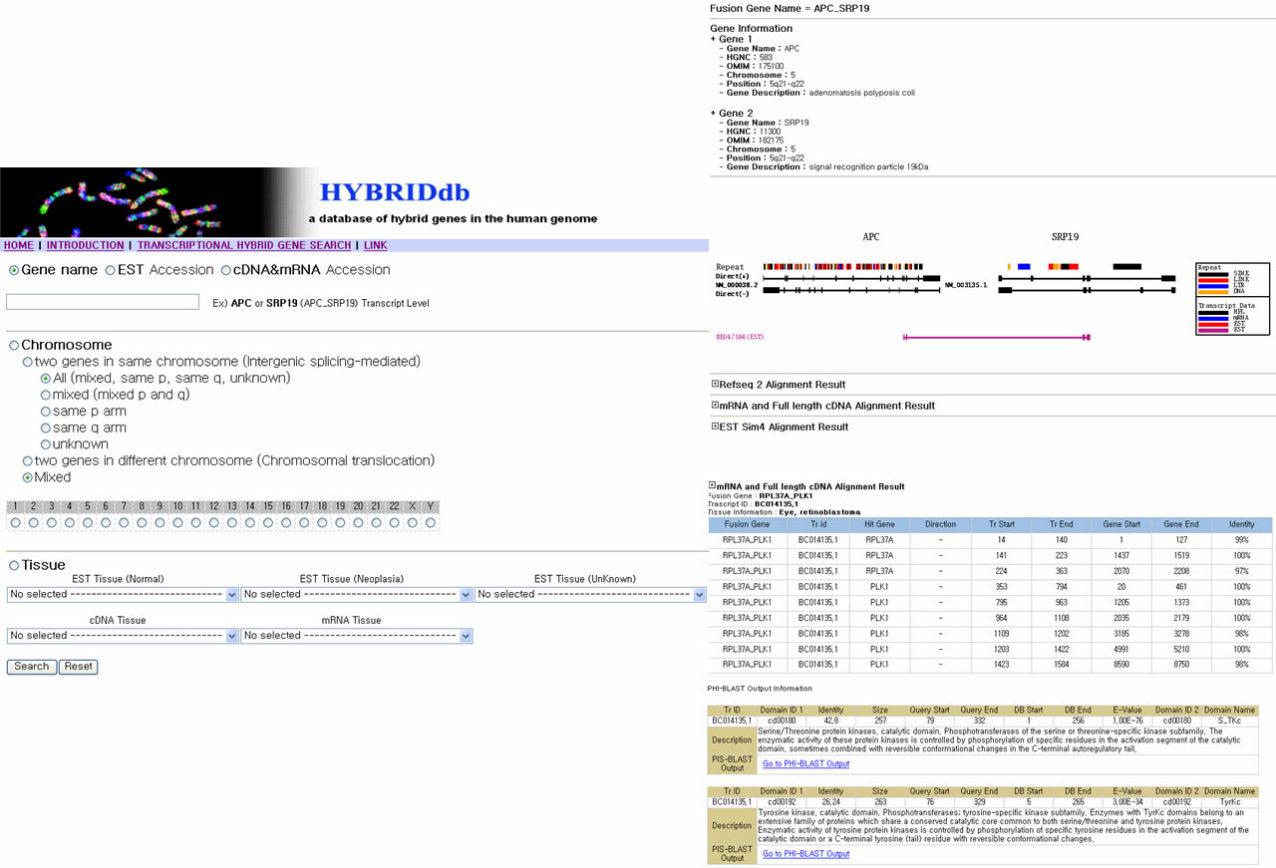
A snapshot of HYBRIDdb interface. The hybrid genes web retrieval interface and a sample from the results pages of HYBRIDdb. The web interface provides access to the database contents in three ways. The results pages are listed in a tabular format which provides evidence for hybrid formation, as well as information about the alignment of hybrid gene pairs within the human genome. The graphic viewer shows hybrid gene formation events that are represented by the exon-intron splicing structure of mRNAs/ESTs/cDNA. And also, the results page includes transcript information about the hybrid gene pairs and tissue, pathology, and organ information about the target gene.

HYBRIDdb is a biological database that uses a MySQL management system to transfer the data from a primary database. The HYBRIDdb database can be accessed through a CGI-Python base web interface that includes one retrieval section, referred to as the transcriptional hybrid genes section (Figure 2). HYBRIDdb provides detailed information regarding the exploration of a specific hybrid gene of interest.

### Transcriptional hybrid genes interface

The transcriptional hybrid gene was created by joining the transcript portions of two different genes in the human genome. Access to the database can be obtained in three ways. First, users may search for genes of interest using the HUGO symbol, and will retrieve sequences and detailed information from the NCBI data bank. Secondly, users may search interesting gene names by clicking on a list of genes on the view page according to their chromosome numbers. Moreover, it is possible for users to view the results of this search by clicking on genomic loci. Thirdly, users can view the results of this search by clicking on pathology information or tissue information, and they can also acquire mRNA sequences from the NCBI data bank for further study.

The graphic viewer shows hybrid gene events in the human genome that are represented by the exon-intron splicing structure of mRNAs/ESTs, and functional analysis from the conserved domain database using RPS-BLAST [26]. The results page also includes tissue, pathology, and organ information about the target gene. Importantly, users can see detailed tissue, pathology, and organ information about the target gene in the table displayed on the results page.

## Conclusion

HYBRIDdb is an integrated database for genome-wide hybrid genes in humans. This system can identify hybrid genes containing hybrid transcripts created by chromosomal-mediated translocation and intergenic splicing-mediated gene fusion. HYBRIDdb is constantly being updated with new human gene databases from available sources. We also plan to supplement this database with hybrid genes from other mammalian species so that they can be directly compared with hybrid genes from humans. Our work should provide insight into roles for hybrid genes in human evolution and disease.

## Availability and requirements

HYBRIDdb is publicly available at the URL . Questions and comments are welcomed through the site.

## Abbreviations

BLAST – Basic Local Alignment Search Tool

CGI – Common Gateway Interface

EST – Expressed Sequence Tag

HUGO – Human Genome Organization

INSDC – International Nucleotide Sequence Databases

NCBI – National Center for Biotechnology Information

RPS-BLAST – Reversed Position Specific Blast

## Authors' contributions

DS Kim analyzed contents of this paper and wrote the manuscript. JW Huh contributed the manuscript correction and continuous discussion. HS Kim participated in its analysis and provided essential direction.
